# Beneficial Effects of Time-Restricted Eating on Metabolic Diseases: A Systemic Review and Meta-Analysis

**DOI:** 10.3390/nu12051267

**Published:** 2020-04-29

**Authors:** Shinje Moon, Jiseung Kang, Sang Hyun Kim, Hye Soo Chung, Yoon Jung Kim, Jae Myung Yu, Sung Tae Cho, Chang-Myung Oh, Tae Kim

**Affiliations:** 1 Department of Internal Medicine, College of Medicine, Hallym University, Chuncheon 24252, Korea; sinjei1129@gmail.com (S.M.); soo3802@hanmail.net (H.S.C.); eun99star@naver.com (Y.J.K.); jaemyungyu@hallym.or.kr (J.M.Y.); 2 Department of Biomedical Science and Engineering, Gwangju Institute of Science and Technology, Gwangju 61005, Korea; wltmd1006@gist.ac.kr (J.K.); tjdfud5759@naver.com (S.H.K.); 3 Department of Urology, College of Medicine, Hallym University, Chuncheon 24252, Korea; cst326@paran.com

**Keywords:** time-restricted eating, circadian rhythm, obesity, metabolic syndrome, meta-analysis

## Abstract

Various behavioral and physiological pathways follow a pre-determined, 24 hour cycle known as the circadian rhythm. Metabolic homeostasis is regulated by the circadian rhythm. Time-restricted eating (TRE) is a type of intermittent fasting based on the circadian rhythm. In this study, we aim to analyze systemically the effects of TRE on body weight, body composition, and other metabolic parameters. We reviewed articles from PubMed, EMBASE, and the Cochrane Library to identify clinical trials that compared TRE to a regular diet. We included 19 studies for meta-analysis. Participants following TRE showed significantly reduced body weight (mean difference (MD), −0.90; 95% confidence interval (CI): −1.71 to −0.10) and fat mass (MD: −1.58, 95% CI: −2.64 to −0.51), while preserving fat-free mass (MD, −0.24; 95% CI: −1.15 to 0.67). TRE also showed beneficial effects on cardiometabolic parameters such as blood pressure (systolic BP, MD, −3.07; 95% CI: −5.76 to −0.37), fasting glucose concentration (MD, −2.96; 95% CI, −5.60 to −0.33), and cholesterol profiles (triglycerides, MD: −11.60, 95% CI: −23.30 to −0.27). In conclusion, TRE is a promising therapeutic strategy for controlling weight and improving metabolic dysfunctions in those who are overweight or obese. Further large-scale clinical trials are needed to confirm these findings and the usefulness of TRE.

## 1. Introduction

All organisms have evolved to survive in the changing environment of the 24 hour light–dark cycle. The time to eat and sleep can be crucial factors for survival. Various behavioral and physiological pathways are constrained to periods of approximately 1 day, following a cycle known as the circadian rhythm.

In mammals, the suprachiasmatic nucleus (SCN) of the hypothalamus, which contains approximately 20,000 neurons, is the primary generator of endogenous circadian rhythms and functions as a master clock to regulate the peripheral systems. The transcription–translation feedback loops in the cells establish circadian rhythms by regulating membrane electrical activity and intracellular mechanisms [1,2]. The SCN obtains input from the photosensitive ganglion cells in the retina to entrain the master clock to environmental solar cycles. Outputs from the SCN synchronize the rhythms in other brain regions and in the peripheral organs [3]. The molecular clock loops are also present in cells of the pancreas, liver, adipose tissue, and immune system [4,5,6,7], and feedback loops have crucial roles in peripheral metabolism. Previous studies have reported that dysfunctions in peripheral clock genes can lead to deleterious changes in lipid metabolism [8], glucose homeostasis [9], and inflammatory pathways.

Communication between the master and peripheral clocks operates via rhythmic neuronal and humoral signals. The SCN outputs influence rhythms in other brain regions via neuronal connections. The peripheral clocks are entrained by autonomic innervation and by glucocorticoid hormones from the central nervous system. The hypothalamus obtains humoral signals from peripheral organs in the form of gut-derived ghrelin, glucagon-like peptide-1 (GLP-1), and adipocyte-derived leptin [10,11,12,13,14]. The SCN also appears to directly accept input signals from peripheral organs, given that receptors for metabolic hormones such as insulin, ghrelin, and leptin are expressed in the SCN [15,16,17]. While the SCN is primarily synchronized by light, peripheral clocks are mainly dependent on feeding time. Feeding–fasting cycles are the major synchronizers for peripheral oscillators. Thus, irregular feeding times can induce a shift in the peripheral clock and internal desynchronization through the decoupling of peripheral clocks, resulting in health consequences such as metabolic syndrome. This type of internal desynchronization has been shown to cause higher levels of postprandial glucose intolerance and decreased insulin sensitivity to glucose [18]. Prolonged desynchrony between the endogenous clock and feeding/fasting cycles can cause the impairment of *β* cell function [9,19,20,21,22]. Therefore, the concept of timed food intake in coordination with the circadian rhythm has received medical attention; thus, the therapeutic effects of time-restricted feeding on metabolic syndrome have been studied.

Eating strategies to prevent obesity and metabolic syndrome are classified into three types: calorie restriction (CR), intermittent fasting (IF), and time-restricted eating (TRE). CR involves a 25% reduction in the daily caloric intake with the usual timing of mealtimes. In IF, the eating period is restricted, with an unintentional reduction in calorie intake. TRE involves consuming all calories within a consistent 8–12 h daily timespan. While IF emphasizes the ratio of fasting/feeding durations, TRE emphasizes the timing of eating within a limited duration without involving CR. As the timing of eating is essential to synchronize the peripheral clock to the central clock, the restriction of the timing and duration of eating may reduce desynchronization between the central and peripheral clocks and recover impaired metabolic pathways. Thus, TRE may be a more effective strategy for combating metabolic syndrome. Recent papers based on small-scale pilot studies reported that TRE reduced body weight and the risks of metabolic diseases and showed beneficial effects on metabolic dysfunctions [23]. However, no prospective large-scale study has been conducted on the benefits of TRE. A meta-analysis provides objective and strong integrative evidence in medical research [24]. In this study, we aim to investigate the effects of TRE intervention on metabolic parameters such as body weight, glucose metabolism, blood pressure, and lipid profiles in adults via a systemic review and meta-analysis.

## 2. Materials and Methods

### 2.1. Search Strategy

The literature search was conducted as per the recommended protocol by the Preferred Reporting Items for Systematic Reviews and Meta-Analyses (PRISMA) (Supplementary Table S1). Data were extracted by two investigators (S.M. and C.-M.O). Before the data extraction, two reviewers (S.M., C.-M.O.) refined the data extraction tables. S.M. and C.-M.O. searched citation databases, including PubMed, EMBASE, and the Cochrane Library, using the same terms on the same day and crosschecked for accuracy (inception to April 3, 2020). S.M. and C.-M.O. extracted data independently using the predefined tables for data extraction. Discrepancies were resolved by discussion with a third investigator (T.K.). In addition, we conducted Google searches for gray literature. No language restrictions were placed on the searches. Search terms included combinations of “time restricted feeding”, “time restricted diet”, or “time restricted eating”, and “weight”, “obesity”, “blood pressure”, “hypertension”, “insulin”, “glucose”, “diabetes”, “cholesterol”, “triglycerides”, or “dyslipidemia” (Supplementary Table S2).

### 2.2. Study Selection

We included studies with the following characteristics: (1) Population: adults aged 20 years or older; (2) Intervention: a daily fasting period of 12–20 h; (3) Comparators: control group in randomized controlled trials (RCTs) or non-randomized controlled trials, or subjects before TRE intervention in studies with a one group pretest–posttest design; (4) Outcomes: data on changes in at least one of the following: weight, blood pressure, hypertension, insulin, glucose, total cholesterol, triglycerides, low-density lipoprotein cholesterol (LDL-C), or high-density lipoprotein cholesterol (HDL-C); (5) Study design: clinical trials using TRE. We defined TRE intervention as fasting for 12–20 h because generally people eat 3 times a day over a 12 hour period.

**Figure 1:**
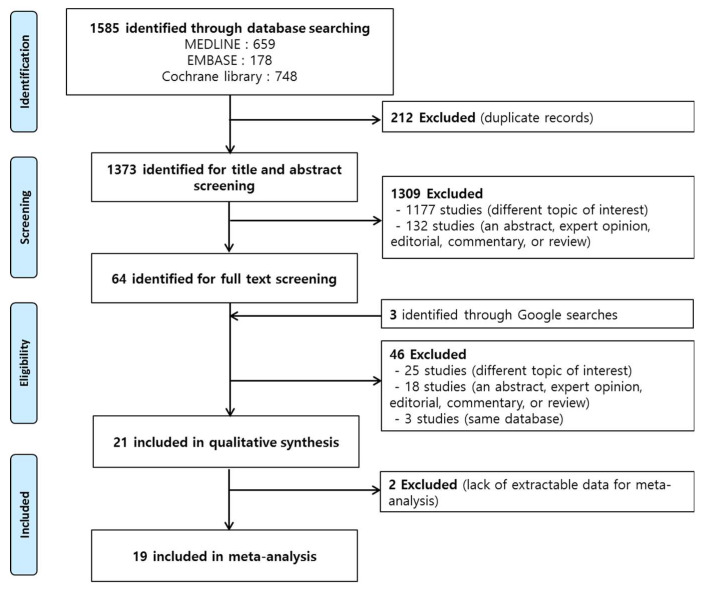
Schema of the search strategy.

We excluded studies with the following characteristics: (1) articles on animal studies or in vivo experiments, only abstracts, and non-original articles including expert opinions or reviews; (2) studies with insufficient information on the TRE regimen; (3) studies including intermittent or periodic fasting/energy restriction; (4) studies on religious fasting including Ramadan fasting; and (5) studies including participants with acute or chronic diseases, such as gastrointestinal diseases or cancer, that affected the outcomes. Based on these criteria we excluded 1569 studies and, finally, performed meta-analysis using 19 studies (Figure 1).

### 2.3. Data Extraction

The following variables were independently extracted by these investigators using the same criteria: first author, publication year, characteristics of the participants, number of study participants, mean age, sex, anthropometric data, body composition, blood pressure, fasting glucose concentration, and lipid profile.

### 2.4. Quality Assessment

We used the “Revised Cochrane risk-of-bias tool for randomized trials (ROB-2.0)” tool to assess the quality of RCTs and “Risk Of Bias in Non-randomized Studies of Interventions (ROBINS-I)” tool to assess non-randomized studies [25]. Discrepancies were resolved by discussion with a third investigator (T.K.).

### 2.5. Data Analyses and Statistical Methods

The pooled effect sizes were presented as mean differences (MD) and 95% confidence interval (CI) using the mean with standard deviation (SD) values before and after TRE intervention considering the heterogeneity of study participants and measurement of outcomes. Inter-study heterogeneity was tested using Cochrane Q statistic and quantified by Higgins I^2^ statistic. An I^2^ statistic of 50% or higher indicated heterogeneity. Publication bias was examined with a funnel plot and Egger’s test, and sensitivity analysis was performed. Considering the heterogeneity of study participants, subgroup analysis was performed by metabolical health status. All analyses were conducted using Comprehensive Meta-Analysis software version 3 (Biostat, Englewood, NJ, USA).

## 3. Results

### 3.1. Study Characteristics

The literature search yielded 1585 (PubMed: 659, EMBASE: 178, Cochrane Library: 748) articles, after excluding 212 duplicate studies. After excluding articles that did not meet the inclusion criteria, 1373 studies were assessed for eligibility. Upon further review and quality assessment, we found 21 studies that met the topic of interest. Among the 21 studies [23,26,27,28,29,30,31,32,33,34,35,36,37,38,39,40,41,42,43,44,45], two were excluded for meta-analysis [26,27]. Their main results have been summarized in Supplementary Table S3. Finally, 16 studies from a database search and 3 studies from a Google search were included in the meta-analysis (11 RCTs, 2 non-randomized controlled trials, 1 historically controlled trial, 5 trials with one group pretest–posttest design) (Figure 1).

A total of 475 participants were included in this study, 219 of whom were men. There were 10 studies on healthy individuals and 9 studies on participants with metabolic abnormality (eight studies on patients with overweight/obesity, prediabetes, or metabolic syndrome, and one study on patients with non-alcoholic fatty liver disease). The demographics and clinical characteristics of the participants are summarized in Table 1.

### 3.2. Quality Assessment

**Figure 2:**
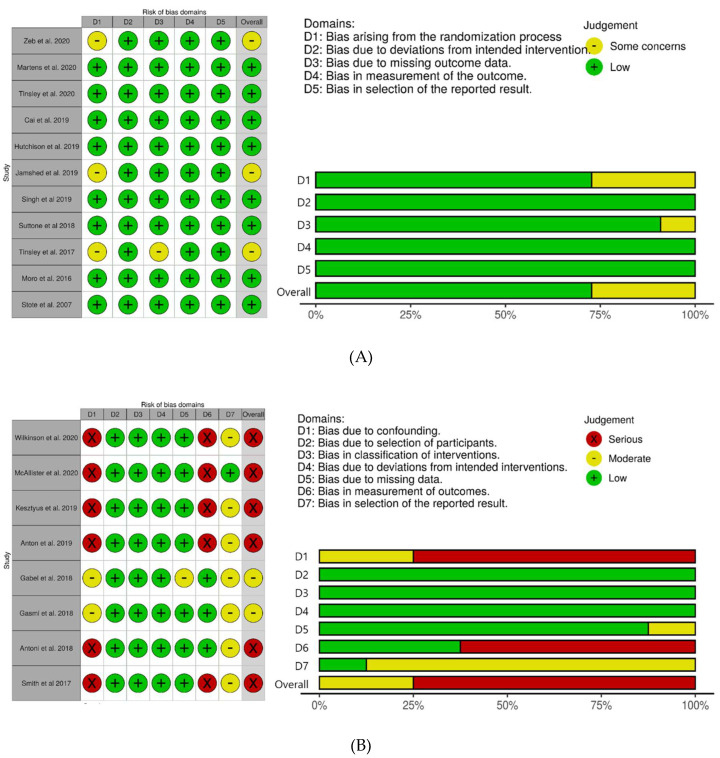
Risk-of-bias assessment in the studies included in the meta-analsysis. (**A**): RCT, (**B**): non-randomized studies.

The risk of bias for the RCTs is reported in Figure 2A. Eight studies had low risk of bias and three studies had some concern of the risk of bias. Two RCTs had some concerns of bias due to the lack of data about the randomization process [28,36]. One RCT had some concerns of bias arising from the randomization procedure and missing outcome data [39]. The risk of bias for the non-randomized trials is shown in Figure 2B. Two of three non-randomized controlled trials had the moderate risks of bias [37,39] and one had the serious risk [44]. Five trials with a one group pretest–posttest design had a serious risk of bias [23,29,32,34,42].

### 3.3. Effect of TRE on Obesity and Body Composition

**Figure 3:**
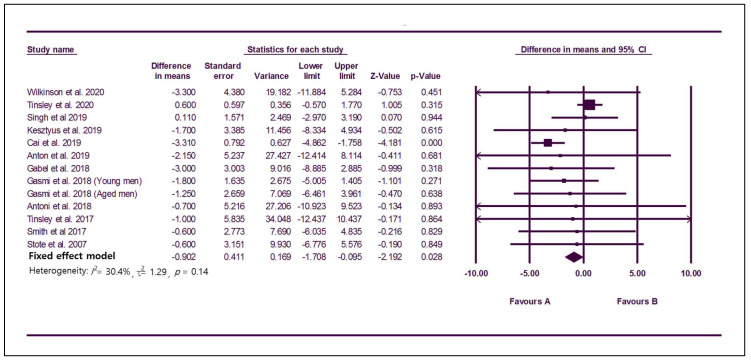
Forest plots summarizing the effect of TRE on body weight compared to baseline.

Twelve studies with 294 participants reported changes in weight with TRE (Figure 3). The MD using a fixed effect model was −0.90 (95% CI: −1.71 to −0.10), which indicates significant weight loss, and the I^2^ was 30.4%, showing no serious heterogeneity among studies (Figure 3). The funnel plot was symmetric, and no publication bias was found (Egger’s test: *p* = 0.14; Supplementary Figure S1A). In the subgroup analysis according to metabolically healthy status, the subgroup with five studies including participants with metabolic abnormality [28,33,34,35,38] showed significant reduction of body weight (MD, −3.19; 95% CI, −4.62 to −1.77; I^2^, 0%) while the subgroup with seven studies including healthy participants [31,38,39,40,42,43,44] did not show significant change of body weight (MD, 0.17; 95% CI, −0.81 to 1.15; I^2^, 0%). In sensitivity analysis, one study [33] showed a significant effect on result. When it was removed, statistical significance disappeared (MD, −0.01; 95% CI, −0.95 to 0.93, Supplementary Figure S2A).

**Figure 4:**
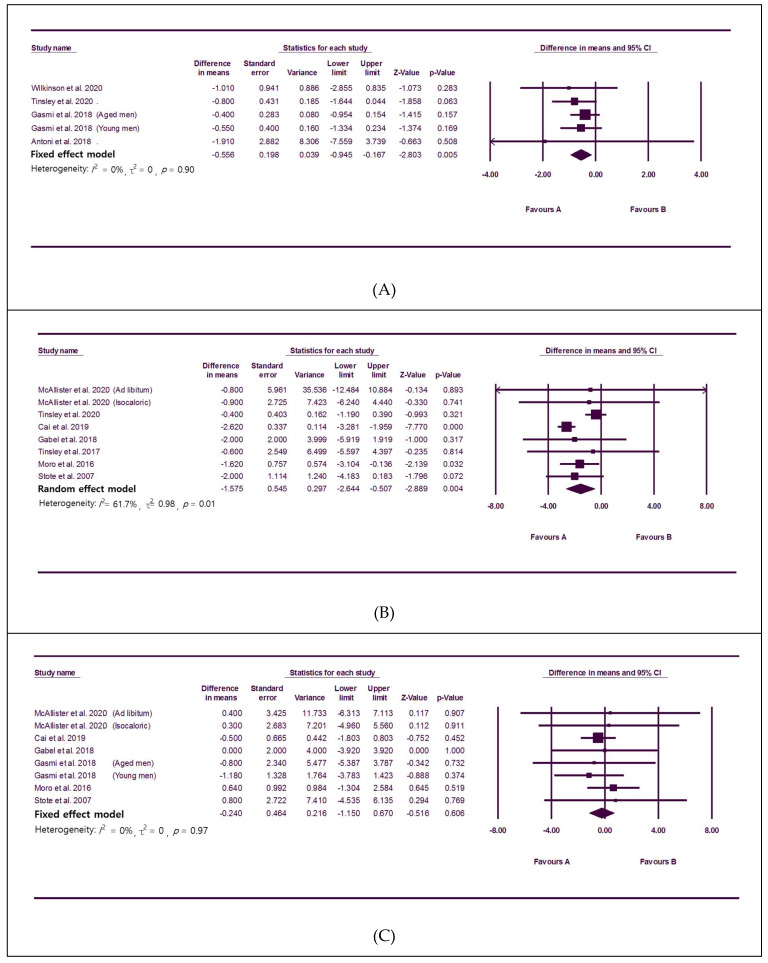
Forest plots summarizing the effect of TRE on body composition compared to baseline; (**A**): fat percent in body, (**B**): total fat mass, (**C**): fat-free mass.

Ten studies [23,29,31,33,37,38,39,40,41,44] with 241 participants reported changes in body composition. TRE significantly lowered fat percentage without significant heterogeneity among studies (MD: −0.56, 95% CI: −0.95 to −0.17, I^2^: 0%; Figure 4A). Fat mass was also significantly reduced (MD: −1.58, 95% CI: −2.64 to −0.51, Figure 4B), and there was heterogeneity among studies (I^2^: 61.7%). Significant publication bias was not observed (Egger’s test: *p* = 0.46; Supplementary Figure S1B). In the subgroup analysis according to metabolically healthy status, the subgroup with five studies including healthy participants [29,31,39,40,41] showed significant reduction of fat mass without heterogeneity (MD: −0.79, 95% CI: −1.44 to −0.14; I^2^, 0%). Among two studies including metabolic abnormality, one [33] showed significant effect of TRE on fat mass reduction. However, the other study [37] failed to show a significant result although TRE tended to lower fat mass. In the sensitivity analysis, one heterogeneous study was identified [33]. When the single outlier study was removed, statistical significance was maintained with the I^2^ reduced to 0% (MD: −0.82, 95% CI: −1.47 to −0.18; Supplementary Figure S2B). There was no significant change in fat-free mass according to TRE (Figure 4C).

### 3.4. Effect of TRE on Cardiometabolic Parameters

Six studies [23,34,37,40,43,45] with 97 participants reported the effect of TRE on blood pressure. TRE significantly lowered systolic blood pressure without significant heterogeneity among studies (Table 2). Significant publication bias was not observed (Egger’s test: *p* = 0.92). Subgroup analysis with four studies [23,34,37,45] including participants with metabolic abnormality showed significant change of systolic blood pressure (MD, −5.42; 95% CI, −9.2 to −1.6; I^2^: 0%; Supplementary Figure S1C). Two studies [40,43] with healthy participants failed to show a significant result, although TRE tended to lower systolic blood pressure. There was no outlier study in sensitivity analysis (Supplementary Figure S2C).

The effect on fasting glucose concentration was reported in 10 studies [23,29,30,31,33,34,36,37,41,44] with 238 participants. TRE significantly lowered fasting glucose concentration (MD, −2.96; 95% CI, −5.60 to −0.33; Table 2) and there was heterogeneity among studies (I^2^: 79.8%). Significant publication bias was not observed (Egger’s test: *p* = 0.73; Supplementary Figure S1D). In the subgroup analysis according to metabolically heatlh status, the subgroup with five studies including participants with metabolic abnormality [23,33,34,36,37] showed significant change of fasting glucose concentration (MD, −2.29; 95% CI, −4.29 to −0.19; I^2^: 2.2%). However, five studies with healthy participants [29,30,31,41,44] did not show significant change although they tended to lower the fasting glucose concentration (MD, −3.65; 95% CI, −7.65 to 0.35; I^2^, 0%). In sensitivity analysis, the statistical significance remained after omitting each study (Supplementary Figure S2D).

Fourteen studies [23,28,29,30,31,32,33,36,37,40,41,44,45] with 343 participants reported changes in lipid profiles with TRE. Triglyceride levels significantly decreased with significant heterogeneity (MD: −11.60, 95% CI: −23.30 to −0.27, I^2^: 81.5%; Table 2). Significant publication bias was not observed (Egger’s test: *p* = 0.28; Supplementary Figure S1E). In subgroup analysis according to metabolically healthy status, there were no significant results in both subgroups (subgroup with five studies including participants with metabolic abnormality [23,32,33,37,45], MD: −7.12, 95% CI: −32.33 to 18.09, I^2^: 81.5%; subgroup with five studies including healthy subjects [28,30,31,40,41], MD: −13.22, 95% CI: −26.94 to 0.50, I^2^: 88.3%). There was no outlier study in sensitivity analysis (Supplementary Figure S2E). There were no significant changes in LDL-C and HDL-C levels (Table 2).

### 3.5. Subgroup Analysis with Controlled Clinical Trials

Subgroup analysis was conducted with controlled clinical trials (11 RCTs and 3 controlled clinical trials without randomization). Although the effect of TRE on the weight change was not significant, TRE reduced fat mass compared to the control group (MD: −1.27, 95% CI: −1.96 to −0.59, I^2^: 0%; Table 3) without heterogeneity among studies. There was no significant change in fat-free mass according to TRE. The effect on fasting glucose concentration was reported in nine studies, and there was no significant change (Table 3). In 10 studies with lipid profiles, there were no significant changes in triglycerides, LDL-C, and HDL-C levels (Table 3). 

## 4. Discussion

To determine the beneficial effects of TRE, we selected 19 clinical studies by a systemic review and performed a meta-analysis. The results of this analysis showed that TRE is an effective treatment strategy for patients who are overweight and obese. Participants using a TRE schedule lost bodyweight and showed a decrease in fat mass. TRE significantly lowered systolic blood pressure and glucose concentration. TRE also changed the lipid profiles of participants. Although LDL-C and HDL-C levels did not show significant differences, triglyceride levels were significantly decreased after TRE.

To overcome the heterogenic nature of our participants’ metabolic profiles (some are healthy young, others are unhealthy obese), we performed subgroup analysis. In the metabolic unhealthy participants, TRE showed a significant reduction in body weight. In the healthy participants, TRE did not show weight reduction, but still showed beneficial effects such as fat mass reduction. This means TRE has beneficial effects on participants with or without metabolic dysfunction.

At the molecular level, circadian rhythms are generated by cell-autonomous transcription–translation feedback loops composed of multiple genes such as *Clock*, *Bmal1*, period (*per*), and cryptochrome (*cry*) [46]. The *Clock*/*Bmal1* heterodimers bind to the E-box in the promoter regions of *per* and *cry*, resulting in the expression of Per and Cry proteins. They also form heterodimers in the cytoplasm to suppress the transcription of *Clock* and *Bmal1* back in the nucleus. *Bmal1* gene expression is activated by retinoic acid receptor-related orphan receptors (RORs) and suppressed by REV-ERBα/β. This negative feedback loop repeats approximately every 24 h, and an abnormal rate of gene expression induces shorter or longer circadian periods [47]. A *Clock* mutation in mice can lengthen the circadian period to as much as 27 h [48].

Mutations in clock genes in peripheral tissues can cause metabolic dysfunctions. Adiponectin and leptin levels are controlled by the molecular circadian rhythm of adipocytes. Mice with adipocyte-specific mutations in the *Clock* gene showed increased body weight, fat composition, and adipocyte size, as well as impaired lipolysis during fasting periods [8]. Intestinal lipogenesis also shows circadian variations, and hypertriglyceridemia and obesity are observed in *Bmal1* knock out mice [9]. Mice with both *Clock* gene mutation and *Bmal1* deletion showed impaired glucose homeostasis with hyperglycemia and hypoinsulinemia [19].

Glucose homeostasis also oscillates in a daily rhythm. Decreased insulin sensitivity and glucose oxidation in the evening are caused by higher levels of postprandial free fatty acid levels in the evening compared to those in the morning [18]. Late lunch eaters show significantly less weight loss than early eaters due to decreased glucose tolerance and insulin functions during the evening [49].

In several murine animal models, feeding time changed the circadian oscillators in the peripheral tissues [50,51]. A recent clinical study also suggested that mealtime might change the central circadian clock [36]. These results emphasize the importance of the “Right Time to Eat” in health. TRE reduces body weight and improves metabolic dysfunction by repairing the disrupted circadian rhythm in people who are overweight and obese [50,52].

TRE is a new treatment strategy for weight control without calorie reduction. This method is a potentially easier way than CR to maintain optimal body weight and health for a longer time because patients do not need to reduce total food intake or to calculate total daily kcal intake. Clinical studies have confirmed the effectiveness of this strategy. Dorothea et al. reported that TRE was predominantly well accepted by participants [32]. In their study, 86% of participants achieved their weight target during the 3 month study period [32]. Research in animal models has shown the beneficial effects of TRE on obesity, diabetes, fatty liver, cardiometabolic dysfunctions, and lifespan [19,46,47,48,49]. Several pilot studies have reported the beneficial effects of TRE in humans [19,27,37,40,41].

Intriguingly, in our study, TRE did not reduce total mass. This implies that TRE reduces fat mass selectively without muscle loss. This result may explain why TRE improves metabolic dysfunction in patients who are overweight or obese, particularly because muscle mass is an important factor in controlling body weight and maintaining a metabolically healthy state [53].

Our data suggest that TRE is a better strategy than CR diet to lose weight. According to a recent review [54], the contribution of fat-free mass loss often exceeded 35% of the total weight loss in persons with normal weight and 20%~30% of the total weight loss in persons who are overweight or obese on a CR diet. Therefore, researchers recommend that persons on a CR diet should increase physical activity and protein intake to preserve muscle mass [54]. Conversely, TRE restored muscle function in *Drosophila melanogaster* (common fruit fly), a species of two-winged flies commonly used for circardian rhythm study, under the conditions of obesity or circadian disruption [55]. Moreover, TRE has not been shown to reduce muscle strength in clinical studies [38,56].

Aging is associated with a gradual loss of fat-free mass and a gain of excess body fat. We refer to this condition involving high fat mass with low fat-free mass as sarcopenic obesity [57]. TRE reduces this ratio; thus, TRE may be a good therapeutic strategy to treat obesity in elderly patients. Animal research has provided evidence on the beneficial effects of TRE. TRE reduced intramuscular fat deposits and suppressed obesity-induced myofibril and mitochondrial defects in *Drosophila* [55]. TRE has been shown to reduce whole-body fat accumulation, improve glucose homeostasis, restore cholesterol homeostasis, and improve muscle function in mouse studies [58,59].

This study had some limitations. First, most included studies had small sample sizes, and several studies had high risk of bias. Therefore, further large studies are necessary to clarify the effect of TRE in the general population. Second, most studies were performed over a short duration. Therefore, we could not analyze the long-term benefits and safety of TRE in terms of cardiovascular disease, type 2 diabetes, and mortality. Third, we could not analyze blood parameters related to obesity and metabolic dysfunction such as adiponectin, leptin, and insulin levels because of lack of data. Last, although TRE is one form of intermittent fasting, we could not compare TRE to other types of intermittent fasting because of lack of data.

In conclusion, TRE significantly reduces body weight and fat and improves metabolic parameters associated with cardiometabolic health. Further studies are needed to confirm the long-term outcomes of TRE.

## Figures and Tables

**Table 1:** Summary of the 19 studies included in the present meta-analysis.

Study [Ref.]	Study Design	Participants	Study Duration	TRE Regimen (Fasting: Feeding)	No. of Total Participants	Age	Sex	Body Composition	Blood Pressure	Fasting Glucose	Lipid Level
Wilkinson et al. [23]	One group pretest–posttest design	Metabolic syndrome (met 3 or more criteria)	12 weeks	14:10	19	59 ± 11.4	13 men, 6 women	Weight: 97.84 ± 19.73 kg WC: 109.14 ± 11.21 Fat (%): 36.62	Systolic BP: 127.88 ± 8.89 mmHg Diastolic BP: 78.47 ± 8.74 mmHg	FBS: 106.72 ± 14.77 mg/dL HbA1c: 5.71% ± 0.45%	TC: 181.42 ± 35.80 mg/dL LDLC: 104.33 ± 32.30 mg/dL HDLC: 47.00 ± 47.00 mg/dL TG:161.16 ± 87.30 mg/dL
Zeb et al. [28]	RCT	Healthy young adults	25 days	16:8	80	22.1 ± 2.1	56 men				
McAllister et al. [29]	One group pretest–posttest design	Physically active college men	28 days	16:8	22	22 ± 2.5	10 men	Weight: 80.25 ± 11.8 kg Fat mass: 26.1 ± 20.6 kg Fat-free mass: 71.6 ± 11.5 kg	Systolic BP: 118.3 ± 9.8 mmHg Diastolic BP: 75.7 ± 8.6 mmHg	FBS: 90.8 ± 10.8 mg/dL	TC: 138.8 ± 17.8 mg/dL LDLC: 83.3 ± 16.4 mg/dL HDLC: 40.7 ± 6.8 mg/dL TG: 80.5 ± 33.4 mg/dL
Martens et al. [30]	RCT	Non-obese healthy adults	6 weeks	16:8	22	67 ± 1	10 men, 12 women	Weight: 70.2 ± 2.8 kg Fat (%): 30.4 ± 1.6	Systolic BP: 122 ± 4 mmHg Diastolic BP: 70 ± 2 mmHg	FBS: 92 ± 1 mg/dL	TC: 199 ± 9 mg/dL LDLC: 116 ± 8 mg/dL HDLC: 63 ± 4 mg/dL TG: 98 ± 7 mg/dL
Tinsley et al. [31]	RCT	Young active female	8 weeks	16:8	13	22.1 ± 2.1	13 women	Weight: 63.8 ± 8.5 kg WC: Fat (%): 28.4 ± 1.5% Fat mass: 18.4 ± 1.5 kg Fat-free mass: 45.5 ± 1.3 kg	Systolic BP: 113 ± 2 mmHg Diastolic BP: 67 ± 1 mmHg	FBS: 89 ± 3 mg/dL	TC: 179 ± 10 mg/dL LDLC: 97 ± 7 mg/dL HDLC: 64 ± 4 mg/dL TG: 88 ± 10 mg/dL
Kesztyüs et al. [32]	One group pretest-posttest design	Metabolic syndrome with abdominal obesity	3 months	16:8	40	49.1 ± 12.4	9 men, 31 women	Weight: 88.8 ± 2.1 kg WC: 106.9 ± 13.3		HbA1c: 5.6% ± 2.9%	TC: 209 ± 46 mg/dL LDLC: 127 ± 43 mg/dL HDLC: 54 ± 15 mg/dL TG: 124 ± 71 mg/dL
Cai et al. [33]	RCT	NAFLD	12 weeks	16:8	95	33.6 ± 6.2	29 men, 66 women	Weight: 74.98 ± 8.02 kg WC: 91.54 ± 4.43 Fat mass: 30.27 ± 3.23 kg lean mass: 44.54 ± 4.08 kg		FBS: 92.2 ± 14.8 mg/dL	TC: 175 ± 59 mg/dL LDLC: 106 ± 34 mg/dL HDLC: 45 ± 17 mg/dL TG: 257 ± 155 mg/dL
Anton et al. [34]	One group pretest–posttest design	Overweight, Older adults	4 weeks	16:8	10	77.1	4 men, 6 women	Weight: 96.96 ± 16.2 kg WC: 109.43 ± 12.9	Systolic BP: 145.9 ± 15.6 mmHg Diastolic BP: 78.1 ± 12.4 mmHg	FBS: 105.6 ± 28.2 mg/dL	
Hutchison et al. [35]	RCT	Obese adults	7 days	15:9	15	59 ± 3	15 men	Weight: 105.7 ± 2.6 kg WC: 115 ± 2 Fat (%): 35.1 ± 1.2 Fat mass: 32.5 ± 1.7 kg lean mass: 62.5 ± 2.3 kg	Systolic BP: 141 ± 3 mmHg Diastolic BP: 87 ± 2 mmHg	FBS: 104.5 ± 1.8mg/dL	
Jamshed et al. [36]	RCT	Overweight adults	4 days	18:6	11	32 ± 7	7 men, 4 women	BMI: 30.1 ± 2.7 kg/m^2^		FBS: 92 ± 5 mg/dL	
Gabel et al. [37]	Historically controlled study	Obese adults	12 weeks	16:8	23	50 ± 2	3 men, 20 women	Weight: 95 ± 3 kg Fat mass: 42 ± 2 kg Lean mass: 50 ± 2 kg	Systolic BP: 128 ± 4 mmHg Diastolic BP: 83 ± 2 mmHg	FBS: 79 ± 4 mg/dL	TC: 177 ± 7 mg/dL LDLC: 108 ± 5 mg/dL HDLC: 48 ± 2 mg/dL TG: 105 ± 11 mg/dL
Gasmi et al. [38]	Non-randomized controlled trial	Healthy young and old men	12 weeks	12:12	20	26.90 ± 1.97(young), 51.60 ± 5.87(old)	20 men (10 young and 10 old)	Weight: 75.80 ± 5.09 kg (young) 77.45 ± 8.53 kg (old) Fat (%): 12.40 ± 1.35 (young) 11.60 ± 0.97 (old) Fat-free mass: 66.38 ± 4.22 kg (young) 68.46 ± 7.47 kg (old)			
Tinsley et al. [39]	RCT	Healthy active men	8 weeks	20:4	10	22.9 ± 4.1	10 men	Weight: 87.4 ± 19.2 kg Fat (%): 18.7 ± 3.8 Fat mass: 17.8 ± 7.8 kg Lean mass: 60.4 ± 11.2 kg			
Stote et al. [40]	RCT	Healthy normal weight adults	8 weeks	20:4	15	45.0 ± 0.7	5 men, 10 women	Weight: 66.5 ± 3.1 kg Fat mass: 16.2 ± 1.2 kg Fat-free mass: 50.1 ± 2.9 kg	Systolic BP: 115.6 ± 4.2 mmHg Diastolic BP: 68.1 ± 2.6 mmHg		TC: 182.0 ± 8.5 mg/dL LDLC: 109.1 ± 8.6 mg/dL HDLC: 53.5 ± 3.9 mg/dL TG: 97.1 ± 86 mg/dL
Moro et al. [41]	RCT	Trained male	8 weeks	16:8	17	29.94 ± 4.07	17 men	Weight: 83.9 ± 12.8 kg Fat mass: 10.9 ± 3.5 kg Fat-free mass: 73.1 ± 5.7 kg		FBS: 96.64 ± 5.1 mg/dL	TC: 193.45 ± 6.6 mg/dL LDLC: 114.58 ± 11.33 mg/dL HDLC: 54.11 ± 5.89 mg/dL TG: 123.78 ± 15.12 mg/dL
Smith et al. [42]	RCT	Healthy young women	4 weeks	16:8	20	21.3 ± 1.2	20 women	Weight: 65.1 ± 12.5 kg Fat mass: 17.7 ± 8.0 kg			
Singh et al. [43]	RCT	Healthy normal weight adults	4 weeks	Single daily meal (morning meal vs. evening meal)	22	30.9 ± 8.95	20 men, 2 women	Weight: 61.91 ± 7.21 kg WC: 83.23 ± 8.35	Systolic BP: 113.1 ± 10.7 mmHg Diastolic BP: 73.8 ± 10.3 mmHg		
Antoni et al. [44]	Non-randomized controlled trial	Healthy middle-aged adults	10 weeks	21:3	13	TRE: 47 ± 3, Control: 45 ± 4	TRE: 1 men, 6 women, Control: 6 women	Weight: 86.2 ± 5.2 kg (TRE), 77.8 ± 7.6 kg (Control) Fat (%): 36.0 ± 2.9 (TRE), 34.6 ± 3.5 (Control)			
Sutton et al. [45]	RCT	Men with prediabetes	5 weeks	18:6	8	56 ± 9	8 men	Weight: 100.7 ± 18.4 kg	Systolic BP: 123 ± 8 mmHg Diastolic BP: 82 ± 7 mmHg	FBS: 102 ± 9 mg/dL	TC: 179 ± 39 mg/dL LDLC: 108 ± 26 mg/dL HDLC: 46 ± 8 mg/dL TG: 120 ± 75 mg/dL

Ref, referenence; No, number; WC, waist circumference; TRE, time-restricted eating; RCT, randomized controlled trial; BP, blood pressure; FBS, fasting blood glucose concentration; TC, total cholesterol; TG, triglycerides; LDLC, low-density lipoprotein cholesterol; HDLC, high-density lipoprotein cholesterol.

**Table 2:** Meta-analyses of the effect of TRE on cardiometabolic parameters compared to baseline.

Outcome	No. of Studies [Reference]	MD (95% CI)	Heterogeneity I^2^ %
Blood pressure			
Systolic blood pressure	6 [23,34,37,40,43,45]	−3.07 (−5.76, −0.37)	0%
Diastolic blood pressure	6 [23,34,37,40,43,45]	−1.77 (−4.51, 1.07)	52.1%
Fasting glucose concentration	10 [23,29,30,31,33,34,36,37,41,44]	−2.96 (−5.60, −0.33)	79.8%
Lipid profile			
Triglycerides	10 [23,28,30,31,32,33,37,40,41,45]	−11.60 (−23.30, −0.27)	81.5%
LDL cholesterol	12 [23,28,29,30,31,32,33,37,40,41,44,45]	0.05 (−4.77, 4.87)	63.8%
HDL cholesterol	11 [23,28,30,31,32,33,37,40,41,44,45]	1.01 (−1.52, 3.55)	62.5%

No, number.

**Table 3:** Meta-analyses with clinical trial studies compared to the control group.

Outcome	No. of Studies [Reference]	MD (95% CI)	Heterogeneity I^2^ %
Weight	9 [31,33,37,38,39,40,43,44,45]	−0.38 (−1.20, 0.29)	0%
Body composition			
Fat percent	4 [31,38,39,44]	−0.55 (−1.15, 0.05)	0%
Fat mass	6 [31,33,37,39,40,41]	−1.27 (−1.96, −0.59)	0%
Fat-free mass	7 [31,33,37,38,39,40,41]	0.07 (−0.70, 0.84)	0%
Fasting glucose concentration	9 [30,31,33,36,37,41,43,44,45]	−0.96 (−4.26, 2.33)	77.4%
Lipid profile			
Triglycerides	9 [28,30,31,33,36,37,40,41,45]	−8.79 (−24.27, 6.90)	80.3%
LDL cholesterol	10 [28,30,31,33,36,37,40,41,44,45]	−1.05 (−8.94, 6.87)	65.9%
HDL cholesterol	10 [28,30,31,33,36,37,40,41,44,45]	2.55 (−1.44, 6.54)	71.1%

## Supplementary Materials

The following are available online at https://www.mdpi.com/2072-6643/12/5/1267/s1, Table S1: Preferred Reporting Items for Systematic Reviews and Meta-Analyses (PRISMA) checklist, Table S2: Electronic search strategy, Table S3: Summary of two studies excluded in the present meta-analysis, Figure S1: Funnel plots, Figure S2: Sensitivity analysis.
