# Supplementary material for: Beneficial Effects of Time-Restricted Eating on Metabolic Diseases: A Systemic Review and Meta-Analysis
Source: Nutrients. 2020 Apr 29;12(5):1267. doi: 10.3390/nu12051267 (PMC7284632; doi:10.3390/nu12051267)

Supplementary Table S1. PRISMA checklist.

Section/topic	#	Checklist item	Reported on page #
**TITLE**			
Title	1	Identify the report as a systematic review, meta-analysis, or both.	1
**ABSTRACT**			
Structured summary	2	Provide a structured summary including, as applicable: background; objectives; data sources; study eligibility criteria, participants, and interventions; study appraisal and synthesis methods; results; limitations; conclusions and implications of key findings; systematic review registration number.	1 (NA because of journal format)
**INTRODUCTION**			
Rationale	3	Describe the rationale for the review in the context of what is already known.	2
Objectives	4	Provide an explicit statement of questions being addressed with reference to participants, interventions, comparisons, outcomes, and study design (PICOS).	2
**METHODS**			
Protocol and registration	5	Indicate if a review protocol exists, if and where it can be accessed (e.g., Web address), and, if available, provide registration information including registration number.	NA
Eligibility criteria	6	Specify study characteristics (e.g., PICOS, length of follow-up) and report characteristics (e.g., years considered, language, publication status) used as criteria for eligibility, giving rationale.	3
Information sources	7	Describe all information sources (e.g., databases with dates of coverage, contact with study authors to identify additional studies) in the search and date last searched.	2-3
Search	8	Present full electronic search strategy for at least one database, including any limits used, such that it could be repeated.	2-3 Figure1 Supplementary table 2
Study selection	9	State the process for selecting studies (i.e., screening, eligibility, included in systematic review, and, if applicable, included in the meta-analysis).	3, Figure1
Data collection process	10	Describe method of data extraction from reports (e.g., piloted forms, independently, in duplicate) and any processes for obtaining and confirming data from investigators.	3, Figure1
Data items	11	List and define all variables for which data were sought (e.g., PICOS, funding sources) and any assumptions and simplifications made.	3
Risk of bias in individual studies	12	Describe methods used for assessing risk of bias of individual studies (including specification of whether this was done at the study or outcome level), and how this information is to be used in any data synthesis.	3
Summary measures	13	State the principal summary measures (e.g., risk ratio, difference in means).	3
Synthesis of results	14	Describe the methods of handling data and combining results of studies, if done, including measures of consistency (e.g., I^2) for each meta-analysis.	3

Section/topic	#	Checklist item	Reported on page #
Risk of bias across studies	15	Specify any assessment of risk of bias that may affect the cumulative evidence (e.g., publication bias, selective reporting within studies).	3, Figure2
Additional analyses	16	Describe methods of additional analyses (e.g., sensitivity or subgroup analyses, meta-regression), if done, indicating which were pre-specified.	3
**RESULTS**			
Study selection	17	Give numbers of studies screened, assessed for eligibility, and included in the review, with reasons for exclusions at each stage, ideally with a flow diagram.	3-4, Figure1
Study characteristics	18	For each study, present characteristics for which data were extracted (e.g., study size, PICOS, follow-up period) and provide the citations.	3,Table1
Risk of bias within studies	19	Present data on risk of bias of each study and, if available, any outcome level assessment (see item 12).	4, Figure2
Results of individual studies	20	For all outcomes considered (benefits or harms), present, for each study: (a) simple summary data for each intervention group (b) effect estimates and confidence intervals, ideally with a forest plot.	8-11, Figure3-4<br>Table2-3
Synthesis of results	21	Present results of each meta-analysis done, including confidence intervals and measures of consistency.	8-11, Figure3-4<br>Table2-3<br>Supplementary figure 1-2
Risk of bias across studies	22	Present results of any assessment of risk of bias across studies (see Item 15).	8-11<br>Supplementary figure 1-2
Additional analysis	23	Give results of additional analyses, if done (e.g., sensitivity or subgroup analyses, meta-regression [see Item 16]).	8-11<br>Table 3<br>Supplementary figure 2
**DISCUSSION**			
Summary of evidence	24	Summarize the main findings including the strength of evidence for each main outcome; consider their relevance to key groups (e.g., healthcare providers, users, and policy makers).	11-12
Limitations	25	Discuss limitations at study and outcome level (e.g., risk of bias), and at review-level (e.g., incomplete retrieval of identified research, reporting bias).	13
Conclusions	26	Provide a general interpretation of the results in the context of other evidence, and implications for future research.	13

FUNDING			
Funding	27	Describe sources of funding for the systematic review and other support (e.g., supply of data); role of funders for the systematic review.	13

Supplementary Table S2. Electronic search strategy.

Database	Search term (inception to April 3, 2020)	Number of Studies
PubMed Title and abstract	#1: ((((Time restricted feeding[Title/Abstract]) OR Time restricted meal[Title/Abstract]) OR Time restricted diet[Title/Abstract]) OR Time restricted fasting[Title/Abstract]) OR Time restricted eating[Title/Abstract]	#1: 1,134
	#2: (((((((((weight[Title/Abstract]) OR obesity[Title/Abstract]) OR cholesterol[Title/Abstract]) OR triglycerides[Title/Abstract]) OR dyslipidemia[Title/Abstract]) OR blood pressure[Title/Abstract]) OR hypertension[Title/Abstract]) OR insulin[Title/Abstract]) OR glucose[Title/Abstract]) OR diabetes[Title/Abstract]	#2: 2,396,450
	#3: #1 AND #2	#3: 659
EMBASE No filters activated	#1: 'time restricted feeding' OR 'time restricted eating' OR 'time restricted meal' OR 'time restricted diet' OR 'time restricted fasting'	#1: 240
	#2: weight OR obesity OR cholesterol OR triacylglycerol OR dyslipidemia OR 'blood pressure' OR hypertension OR insulin OR glucose OR diabetes	#2: 4,491,778
	#3: #1 AND #2	#3: 174
Cochrane Library Trials	#1 time restricted feeding OR time restricted eating OR time restricted meal OR time restricted diet OR time restricted fasting	#1: 991
	#2 weight OR obesity OR cholesterol OR triacylglycerol OR dyslipidemia OR blood pressure OR hypertension OR insulin OR glucose OR diabetes	#2: 308,874
	#3: #1 AND #2	#3: 748

Supplementary Table S3. Summary of Two Studies Excluded in the Present Meta-Analysis.

Study	Study Design	Participants	Study Duration	TRE Regimen (Fasting: Feeding)	No. of Total Participants	Age	Sex	Main Outcome	Reasons to be Excluded from Meta-Analysis	Quality Assessment
Parr et al.	RCT	Overweight/ obese adults	5 days	16:8	11	38±5	11 men	Compared to extended feeding, short-term TRE improved nocturnal glycemic control and was positively perceived in men with overweight/obesity.	Lack of extractable data	Low risk of bias
Ravussin et al.	RCT	Overweight adults	4 days	18:6	11	32±7	7 men, 4 women	TRE did not affect 24-hour energy expenditure and decreased mean ghrelin levels	Lack of extractable data	Low risk of bias

Supplementary Figure S1. Funnel plots. **A**, weight; **B**, fat mass; **C**, blood pressure; **D**, Fasting blood glucose concentration; **E**, triglycerides concentration.

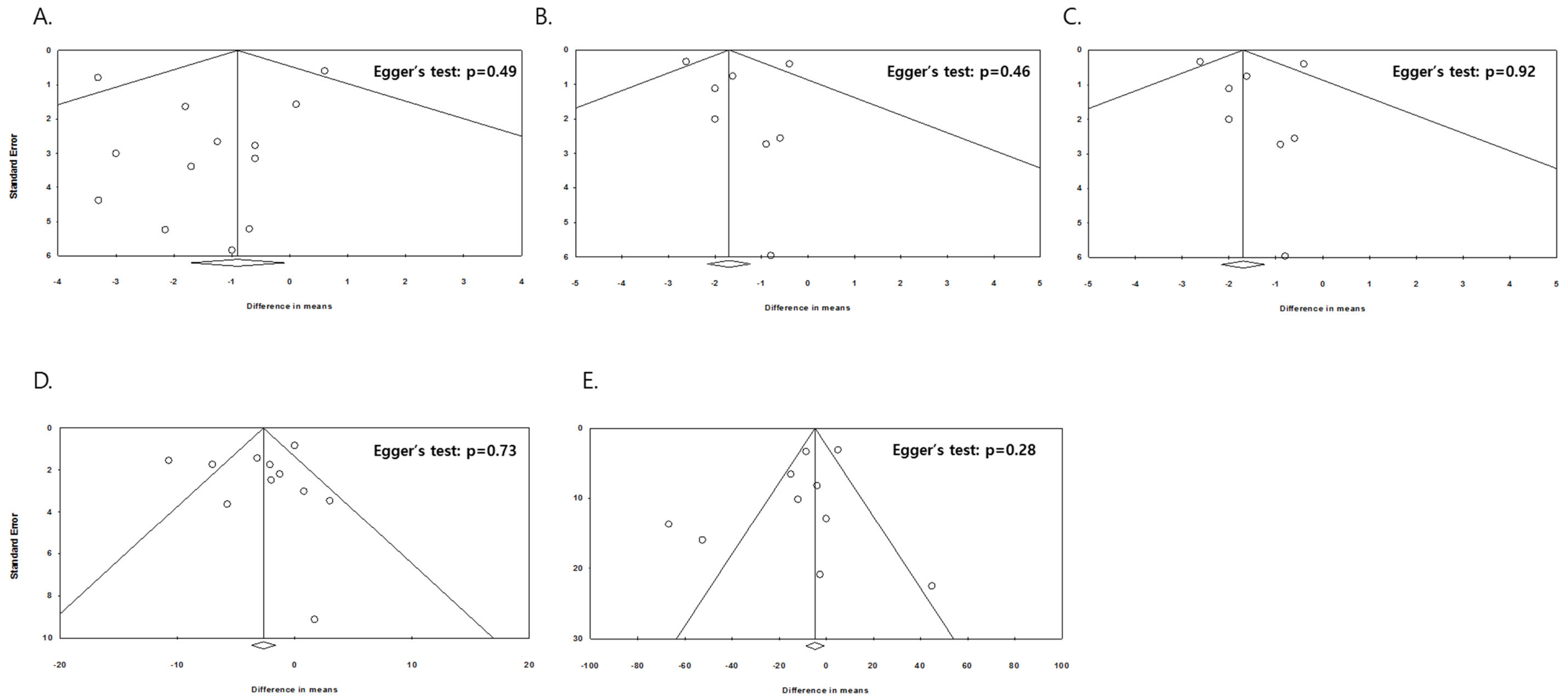

Supplementary Figure S2. Sensitivity analysis. **A**, weight; **B**, fat mass; **C**, blood pressure; **D**, Fasting blood glucose concentration; **E**, triglycerides concentration.

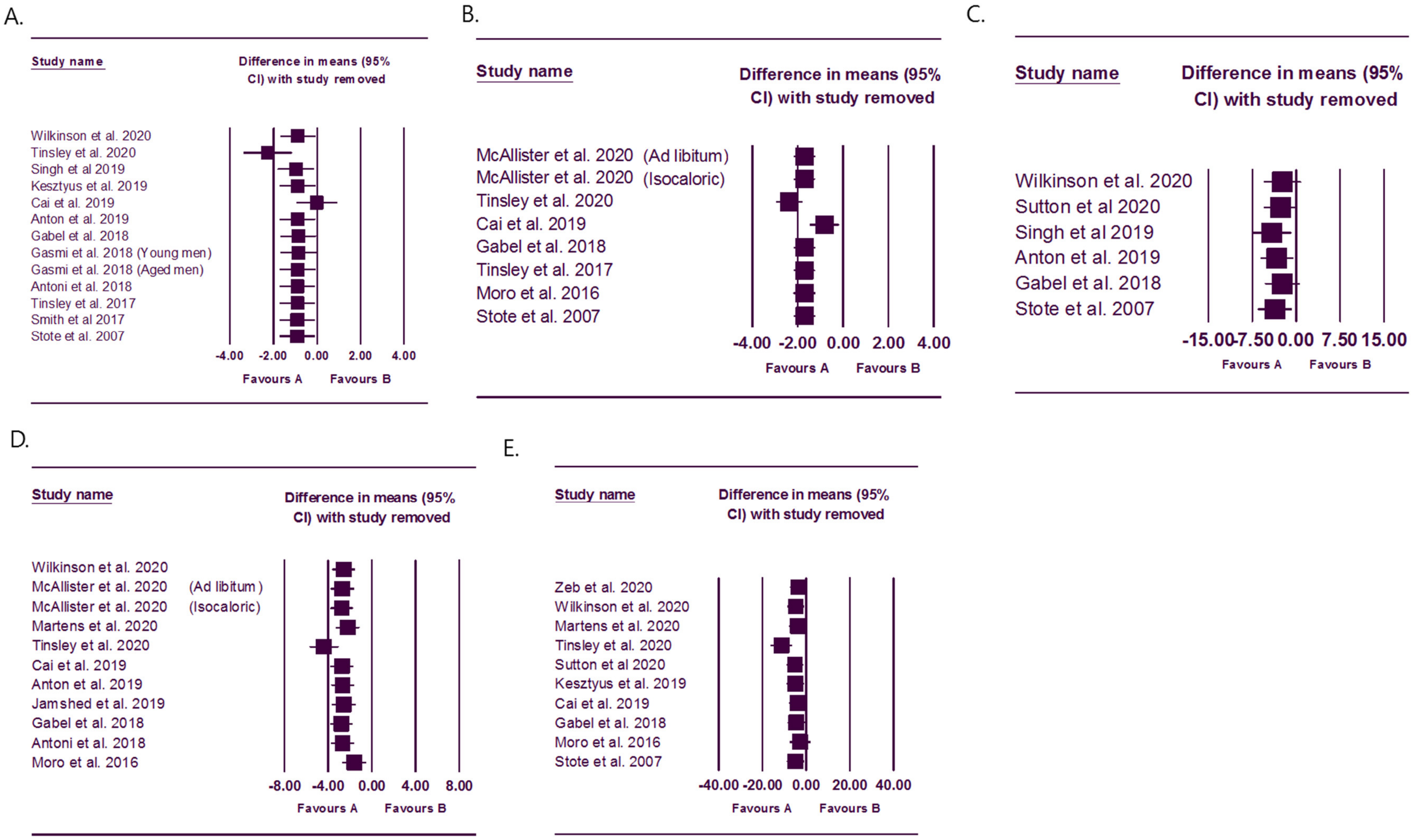

Supplement: Supplementary file 1 [file nutrients-12-01267-s001.pdf]
